# Prevascularized Micro-/Nano-Sized Spheroid/Bead Aggregates for Vascular Tissue Engineering

**DOI:** 10.1007/s40820-021-00697-1

**Published:** 2021-08-18

**Authors:** Maedeh Rahimnejad, Narges Nasrollahi Boroujeni, Sepideh Jahangiri, Navid Rabiee, Mohammad Rabiee, Pooyan Makvandi, Omid Akhavan, Rajender S. Varma

**Affiliations:** 1 Biomedical Engineering Institute, School of Medicine, Université de Montréal, Montreal, Canada; 2 Research Centre, Centre Hospitalier de L’Université de Montréal (CRCHUM), Montreal, Canada; 3 Department of Industrial Biotechnology, National Institute of Genetics and Biotechnology, Tehran, Iran; 4 Department of Biomedical Sciences, Faculty of Medicine, Université de Montréal, Montreal, Canada; 5 Department of Physics, Sharif University of Technology, P.O. Box 11155-9161, Tehran, Iran; 6 Biomaterial Group, Department of Biomedical Engineering, Amirkabir University of Technology, Tehran, Iran; 7 Centre for Materials Interfaces, Istituto Italiano Di Tecnologia, viale Rinaldo Piaggio 34, 56 025 Pontedera, Pisa, Italy; 8 Regional Centre of Advanced Technologies and Materials, Czech Advanced Technology and Research Institute, Palacky University, Šlechtitelů 27, 783 71 Olomouc, Czech Republic

**Keywords:** Vascular tissue engineering, Nano-/micro-sized aggregates, Microspheres

## Abstract

Efficient strategies to promote microvascularization in vascular tissue engineering, a central priority in regenerative medicine, are still scarce; nano- and micro-sized aggregates and spheres or beads harboring primitive microvascular beds are promising methods in vascular tissue engineering. Capillaries are the smallest type and in numerous blood vessels, which are distributed densely in cardiovascular system. To mimic this microvascular network, specific cell components and proangiogenic factors are required. Herein, advanced biofabrication methods in microvascular engineering, including extrusion-based and droplet-based bioprinting, Kenzan, and biogripper approaches, are deliberated with emphasis on the newest works in prevascular nano- and micro-sized aggregates and microspheres/microbeads.

## Highlights

- The first perspective regarding the application of nano-micro size aggregations on the prevascularizations and biomedicine.
- This perspective provides an in-depth discussion regarding the use of these prevascularized nano-micro size aggregates in biomedicine and regenerative medicine.

## Graphical abstract

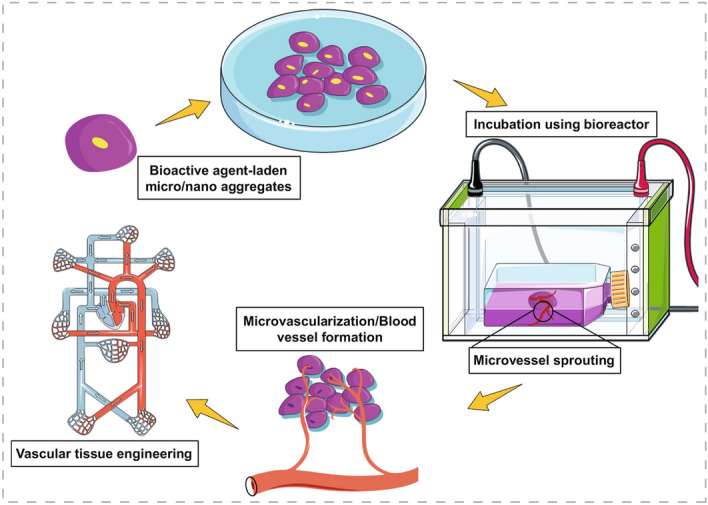

## Vascularization

**Fig. 1:**
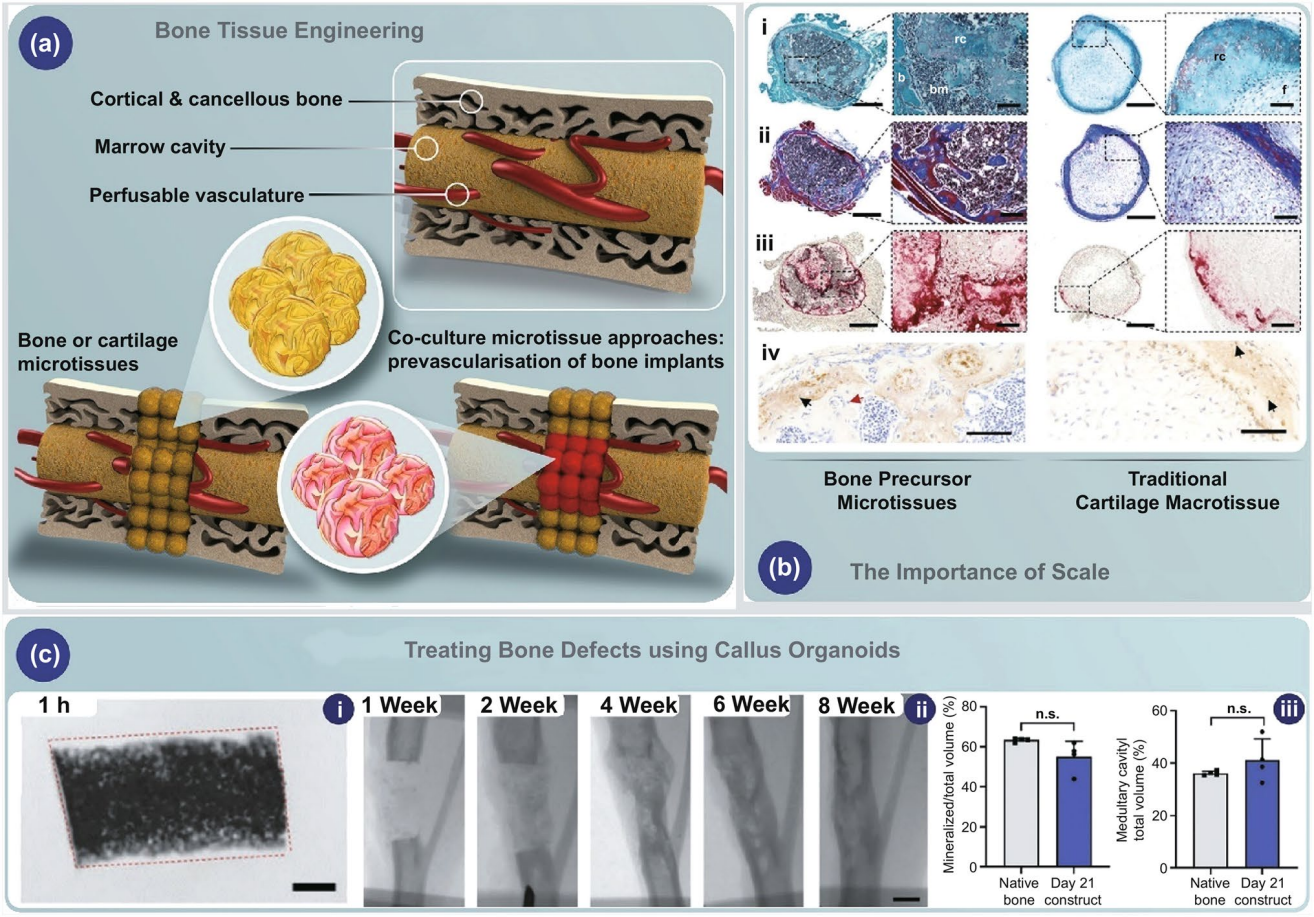
**a** Schematic representation of microtissues in bone tissue engineering. **b** The importance of scale in cartilage precursors for endochondral bone tissue engineering. **c** The same precursor microtissues can be used to heal a murine, critically sized long bone defect. The figure reprinted [18, 19] with permission from Elsevier and Wiley

The biofabrication approach entails building engineered tissues such as vascular networks in the laboratory. In this regard, techniques based on microfluidics technologies and bioprinting can be applied to create vascular networks [1–5]. 3D bioprinting specifically provides a remarkable solution for creating advanced vascularized implants which are difficult to attain via conventional fabrication methods [6–10]. Although these processes have made significant progress, they are not essentially precise and spatiotemporally controllable to simulate the function and physiological complications of the three-dimensional vascular networks [11–13]. In addition to fabrication technique, the most commonly deployed procedure to induce vascularization is the controllable delivery of biomaterials such as hydrogel scaffolds within proangiogenic factors like vascular endothelial growth factor (VEGF) [14–17]. Another approach is cell-based tissue engineering, which have different physicochemical parameters that should be optimized; however, in a specific case, this approach could lead to increasing the cellular density in an specialized organ (Fig. 1) [18].

**Fig. 2:**
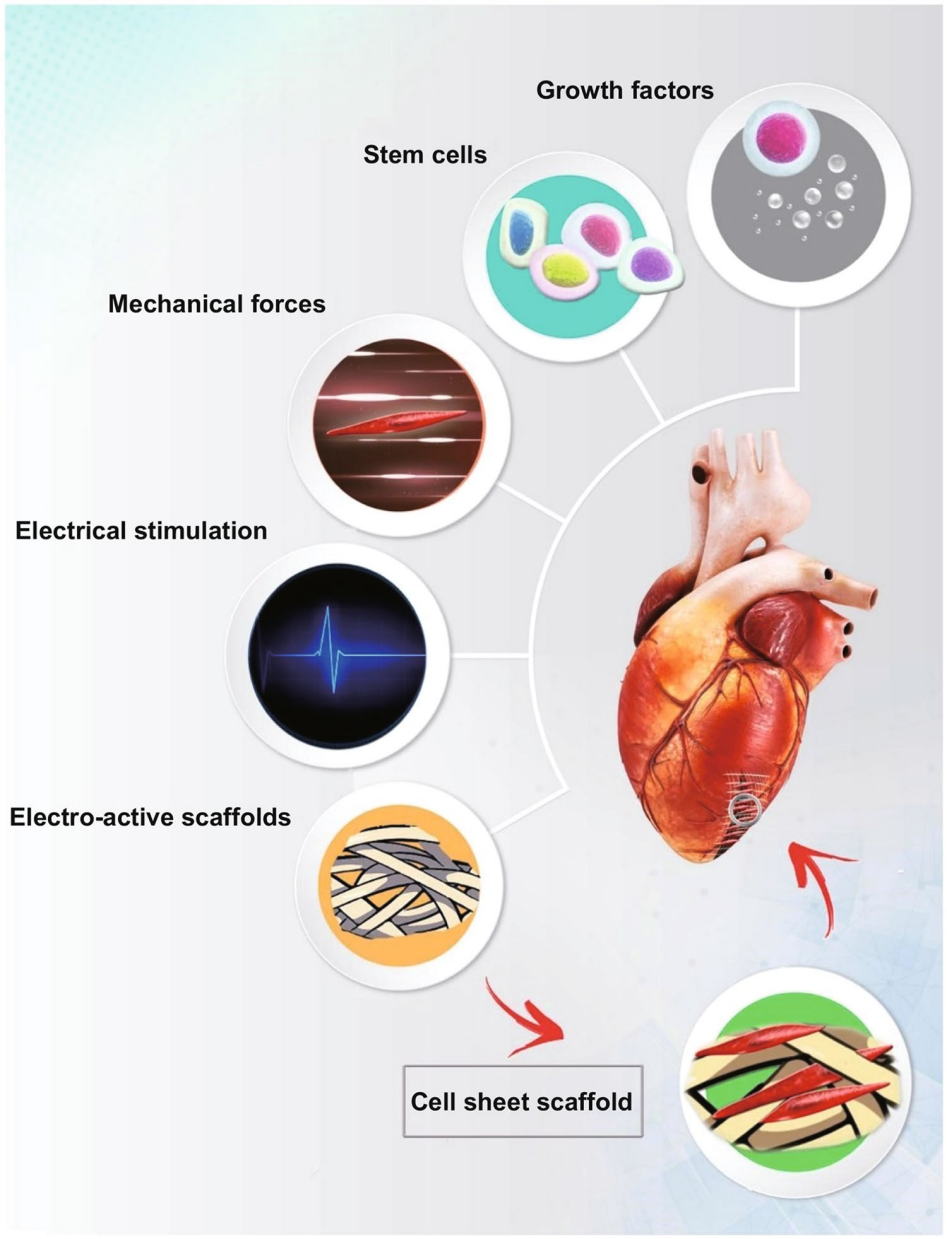
Key factors involved in the cardiac tissue engineering [57]

To engineer vascular tissue, comprehensive understanding of the cardiovascular system is required where capillaries as the smallest type and most numerous blood vessels are distributed densely. They are specialized for regulation of the filtration process to directly exchange nutrients and wastes within tissues which are restricted to 100–200 µm [20–23]. Therefore, engineered tissues that are larger in width or length than these dimensions require innate microvascularization for adequate oxygen and nutrients diffusion and survival in *vivo* [24–26]. Significant impairments in vascularized tissues, for instance bone defects that do not fully heal on their own, are termed critically sized, so physiological regeneration does not occur naturally as a consequence of normal processes eventually leading to chronic pains affecting millions of people throughout the world [27–29]. The limited characteristic healing capacity of large vascular defects and the limitations of current interventions make their treatments a crucial challenge in clinical settings which need to be addressed [22, 27, 28]. The structure of blood vessels is distinct for each of them [30, 31]. Large vessels including arteries and veins are composed of three main microscopic wall layers: an outer layer made up of collagen fibers and elastic tissue, a middle layer encompassing collagen fibers, elastic tissue and smooth muscle, and an inner layer comprising endothelium [32–34]. Arteries contain more percentage of elastic tissue than veins that enables them to enhance their blood conduction capacity as the blood pressure increases [35]. Small vessels including venules arterioles and capillaries have much thinner and narrower walls in proportion to arteries and veins. Venules and arterioles are small diameter blood vessels composed of thin layers of fibrous tissue and smooth muscle, respectively [36–38]. The smallest vessels in the body are capillaries which are normally only one cell in thickness allowing optimal mass exchange and fluid permeability. The cell types vary slightly according to vessel size. Veins, arteries and arterioles consist of endothelial cells (ECs), pericytes and smooth muscle cells (SMCs) [39, 40]. Venules are constituted of endothelial cells (ECs), pericytes and smooth muscle cells (SMCs) which imparts distinguishing characteristics to venules from arteries [41]. Capillaries are made up of single endothelial cells (ECs) and some pericytes which are primarily considered to be cells that stabilize the vessel wall [42]. The main role of large vessels is returning the blood and mass transport toward or away from organs. On the contrary, the small vessels or distinct capillaries are associated with several biological procedures, which include facilitating the transport and absorption of interstitial fluid from the tissues, the migration of lymphocytes, and immune response, etc. [43–45]. Blood vessels function and structures demonstrate their significant complexity. The intricacy of engineering microvasculature is the major challenge in the field of tissue engineering [26, 46, 47]. Also, the key factors for any tissue engineering should be considered as well that includes chemical growth factors [48], stem cells [49], scaffold materials [50], mechanical forces [51], topography of scaffolds [52], electrical stimulation [53], biocompatibility/biodegradability [54], electro-active scaffolds [55] as well as the cell sheet scaffolds [56] (Fig. 2) [57].

### Growth Factors

**Fig. 3:**
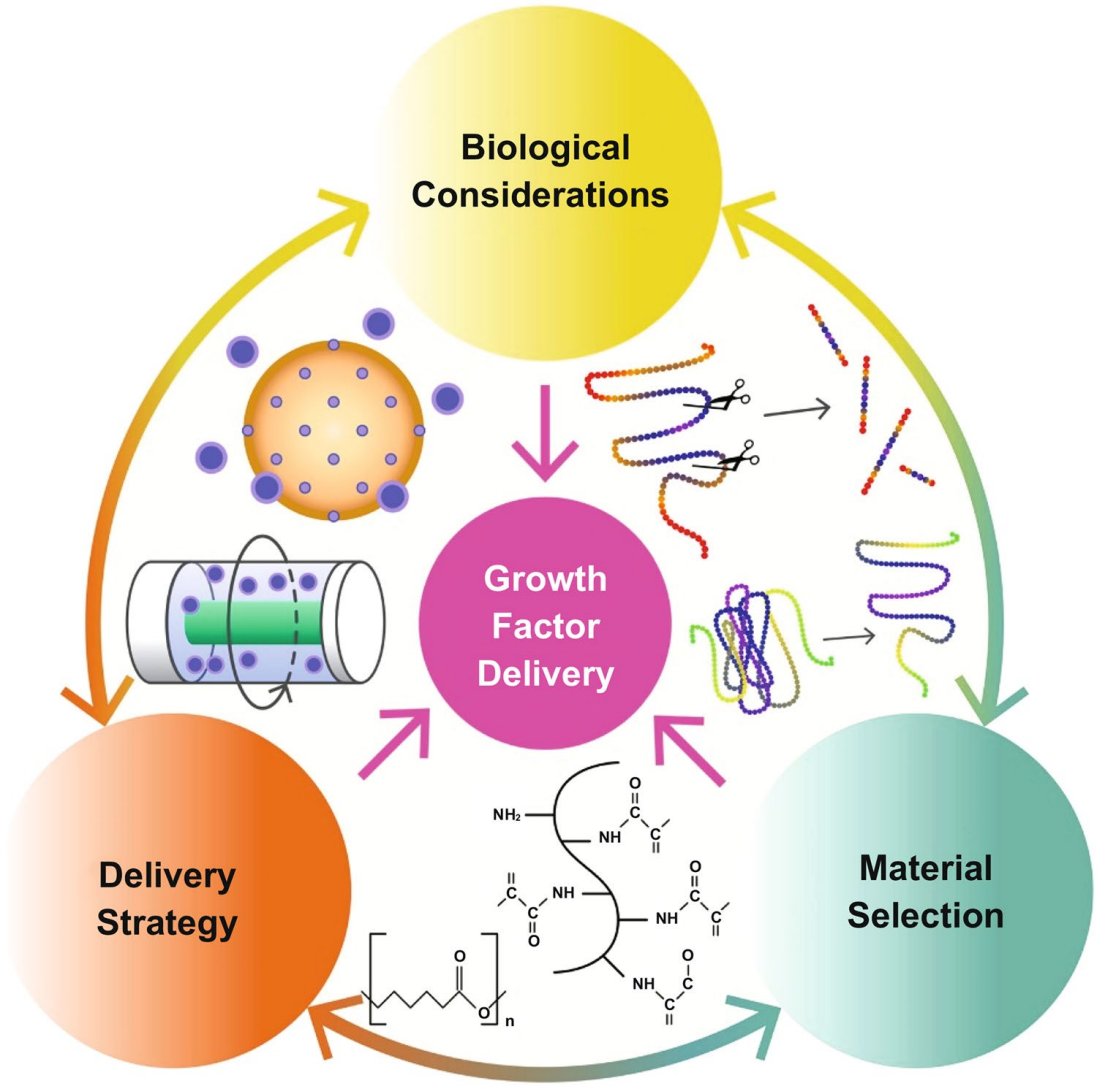
A schematic illustration regarding the delivery of GFs and effect of each key factors on this delivery system [15]

The extracellular matrix (ECM) is a highly dynamic microenvironment that regulates multiple cellular processes to operate mainly by recruitment and activation of different growth factors (GFs). Varying affinities in different locations lead to diverse GF–ECM interactions; also, there are lots of interconnected interactions to the GFs and its ability to have fully functional behavior (Fig. 3) [58–60]. The ECM regulates downstream signaling pathways by GF activity which binds to cellular receptors and leads to the de novo formation of new vessels during angiogenic process. Many in vitro methods have been established to simulate the sprouting through the controllable release of growth factors from biomaterials. In general, deployed growth factors that stimulate the formation of new blood vessels to subtend basic fibroblast growth factor (BFGF), vascular endothelial growth factor (VEGF), platelet-derived growth factor (PDGF), and transforming growth factor-β (TGF-β) [61–64]. Chemical and biological covalent binding reactions can be used to incorporate GFs into matrices or load them into micro-sized aggregates or microspheres for long-term delivery [65–67]. Recently, scientists developed an injectable combination of VEGF-loaded dextran microparticles into poly(lactic-co-glycolic acid) (PLGA) microspheres providing controlled release of GF. The system showed endothelial cells proliferations in vitro and formation of capillaries as well as smooth muscle α-actin-positive vessels in vivo when injected to the rat ischemic tissue [68]. In another study, microfluidic technology was used to create PLGA microspheres consisting of anhydrous reverse micelle (R.M.) dipalmitoylphosphatidylcholine (DPPC) nanoparticles which was loaded with VEGF; slow and sustained release over 28 days was reported resulting in promoted proliferation of human umbilical vein endothelial cells [69]. Similarly, Tayebi research team fabricated PLGA microspheres with average sized of 16–36 µm for controlled release of VEGF [70]. A recent effort reviewed the potential and applications of various micro-sized particles for controlled and sustained GF release [71].

**Fig. 4:**
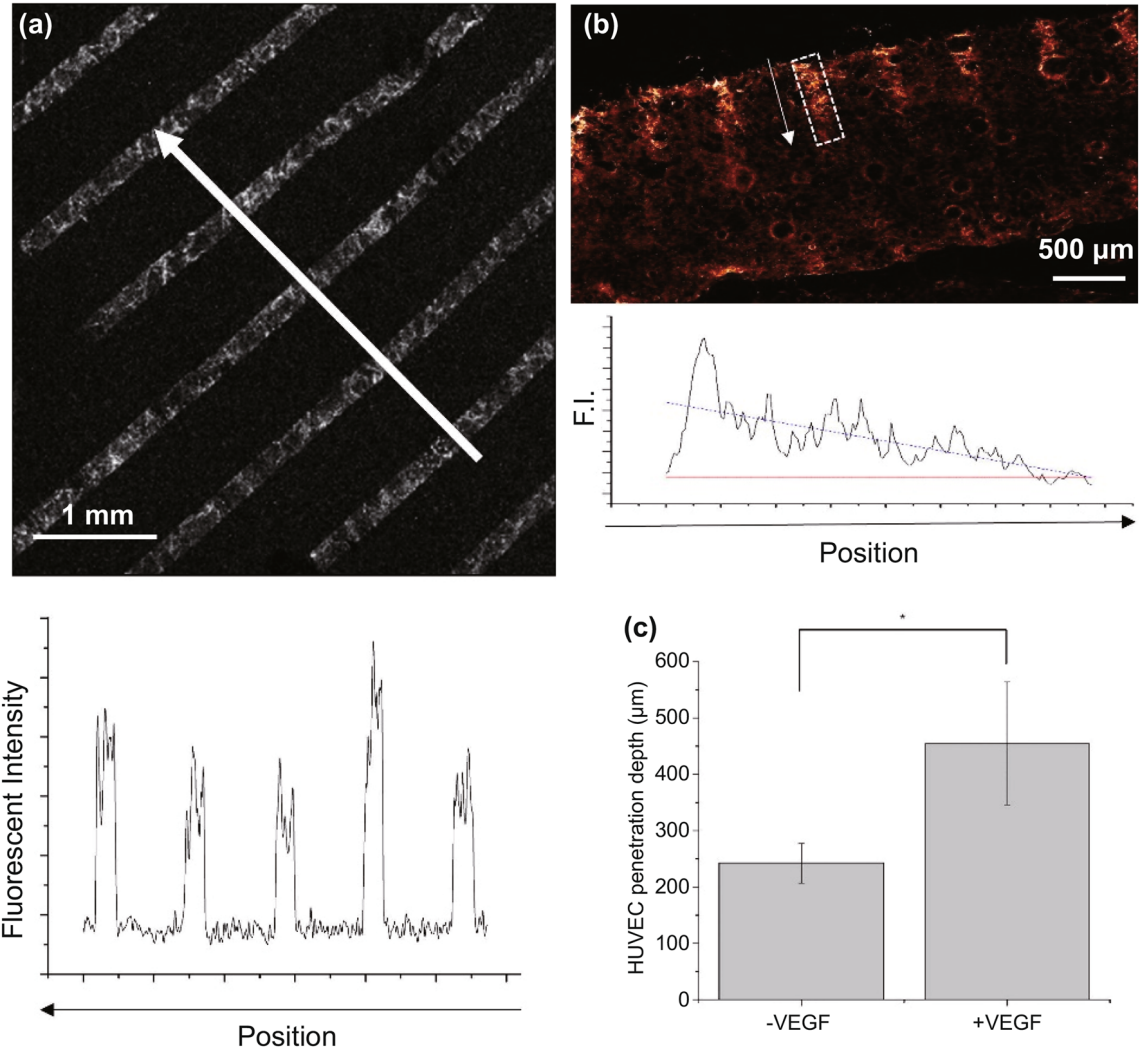
Photopatterning of VEGF onto the surface of collagen-glycosaminoglycan scaffolds; **a** the surface of the collagen-glycosaminoglycan scaffolds photopatterned with VEGFs, **b** cross-sectional microscopy of the photopatterned VEGF onto the surface of collagen-glycosaminoglycan scaffolds, **c** the VEGF bounded amount onto the surface of collagen-glycosaminoglycan scaffolds [80]

**Fig. 5:**
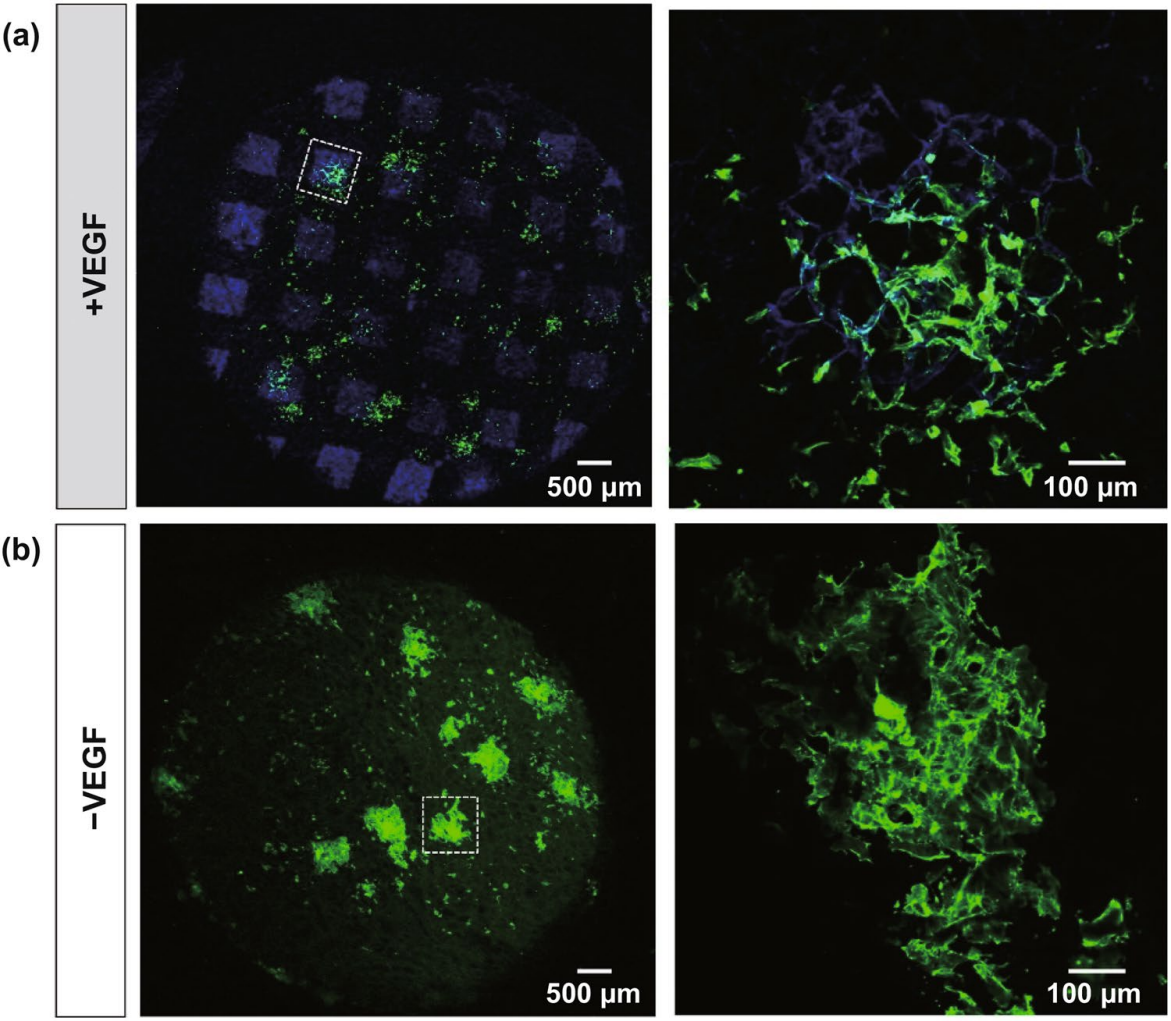
The photopatterned VEGF onto the surface of collagen-glycosaminoglycan scaffolds, **a** images of the HUVEC cells cultured on the modified photopatterned scaffolds, and **b** cultured on the unmodified photopatterned scaffolds [80]

Growth factors can be released only upon a local cellular demand by using various physical stimuli sources that are insignificantly invasive such as light, ultrasound and localized heat [72–76]. For an example, magneto-thermo-responsive smart carrier has been developed using poly(N-isopropylacrylamide) (PNIPAM) to control the release and absorption of VEGF, stimulating human umbilical vein endothelial cells proliferation [77]. Perceptual presentation and gentle release of growth factors has a crucial function in modulating neovascularization [78, 79]. Sprouting of vessels and endothelial cells migration in a specific orientations can be performed by applying light leading to modeling of growth factors, which could lead to a homogenous and aligned patterns of VEGF onto an specific substrate (Fig. 4), and also resulted in high-resolution cellular culturing in the presence of photopatterned VEGFs on the surface of the substrate (Fig. 5) [80, 81]. It has been shown that spatial GF gradient can be removed by interstitial flow with time, which indicates that growth factors and fluid forces collaborate to control the growth of new blood vessels [82].

**Fig. 6:**
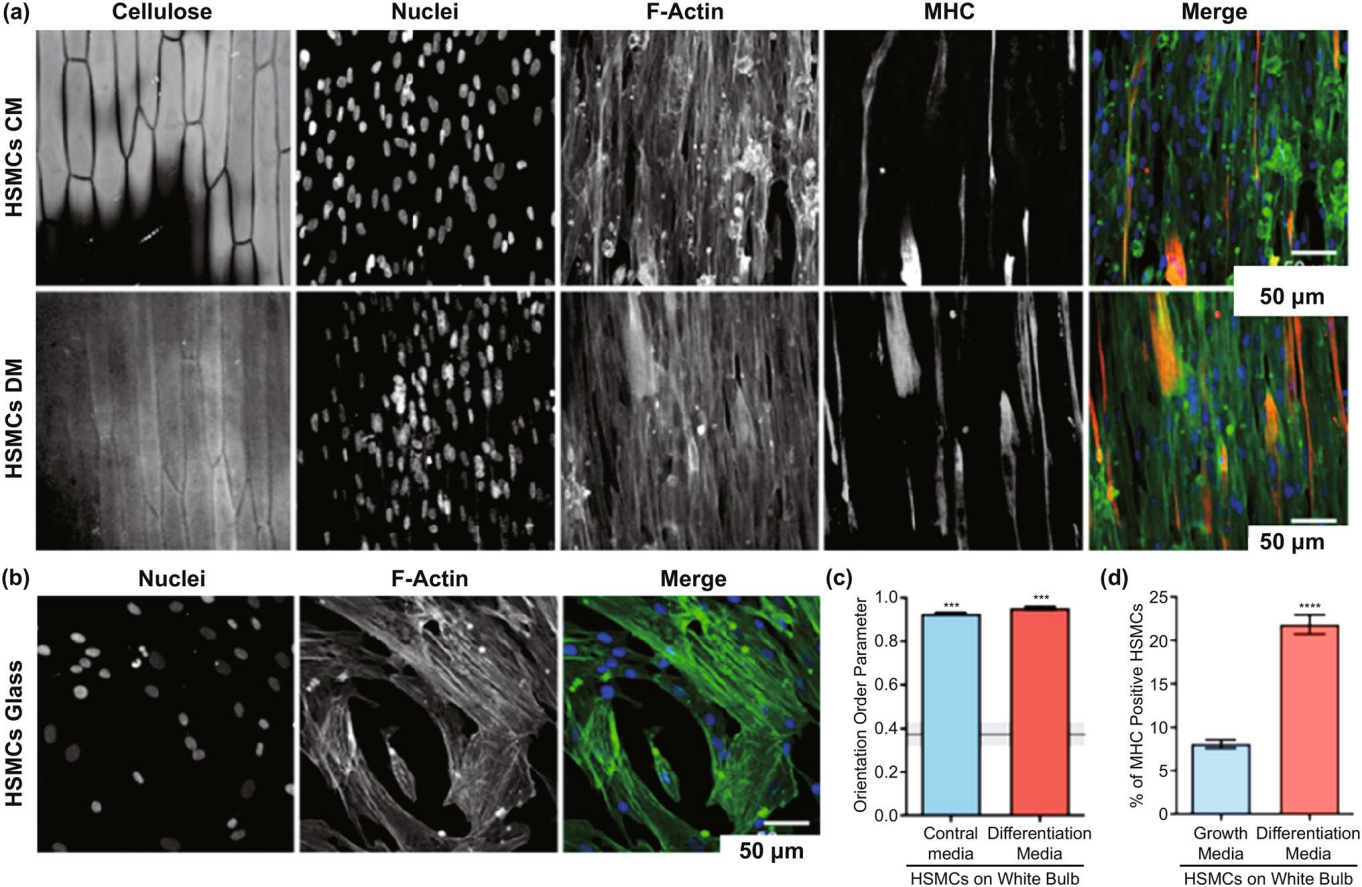
C2C12 cells differentiate into aligned myotubes when cultured on the outer surface of the green onion derived cellulose scaffolds. **a** When cultured on the outer green onion white bulb or green leaf cellulose scaffolds. **b** Example confocal fluorescence image showing C2C12 cells align randomly when cultured on a fibronectin-coated (50 μg mL^−1^) glass coverslip. **c** Quantitative analysis of the fluorescence images obtained for actin alignment is performed via a 2D orientation order parameter (OOP) of C2C12 cells seeded on various substrates. Reprinted with permission from [88]. Copyright 2021 American Chemical Society

Another strategy to model vascularization is via release of proangiogenic paracrine factors by engineering cells function. For instance, scientists demonstrated that incorporation of gelatin microspheres within vascular tissue rings promotes GF release and expression of smooth muscle contractile protein through cellular self-assembly [83]. It has been demonstrated that arteriogenic gene expression profiles are enhanced by preconditioning mesenchymal progenitor cells secreting TGF-β in low concentrations. The secretion of stromal cell-derived factor 1 reportedly promotes the vascular formation when mesenchymal progenitor cells are cultured in infarcted hearts in a myocardial infarction rat model [84]. It has been shown that high-level expression of endogenous VEGF by targeted genetic manipulation of Mesenchymal stem cells (MSCs) significantly improved the angiogenic-supportive capabilities in vivo [85]. An injectable polyethylene glycol-based microbeads modified with VEGF to promote in vivo vascularization resulted in intra-islet engraftment and glucose level regulation [86]. The effective microvascular function is dependent on the fabrication of microvascular mediated with GFs. The lack of these approaches often leads to an out-of-control network organization and the formation of leaky microvessels [87]. Also, another study showed that for skeletal muscle, after culturing the appropriate cells (C2C12 cells) on the growth medium for three days, the culture medium successfully exchanged with myoblast medium, which allows stimulations of cell differentiations as well as the cell alignments. After six days, the procedure was completed and fully cell alignments were observed. An interesting point is about the cells grew on the leaf-derived cellulose scaffolds, which showed fully alignments; however, on the glass coverslips showed partially alignments in some cases (Fig. 6) [88].

### Cell Components

**Fig. 7:**
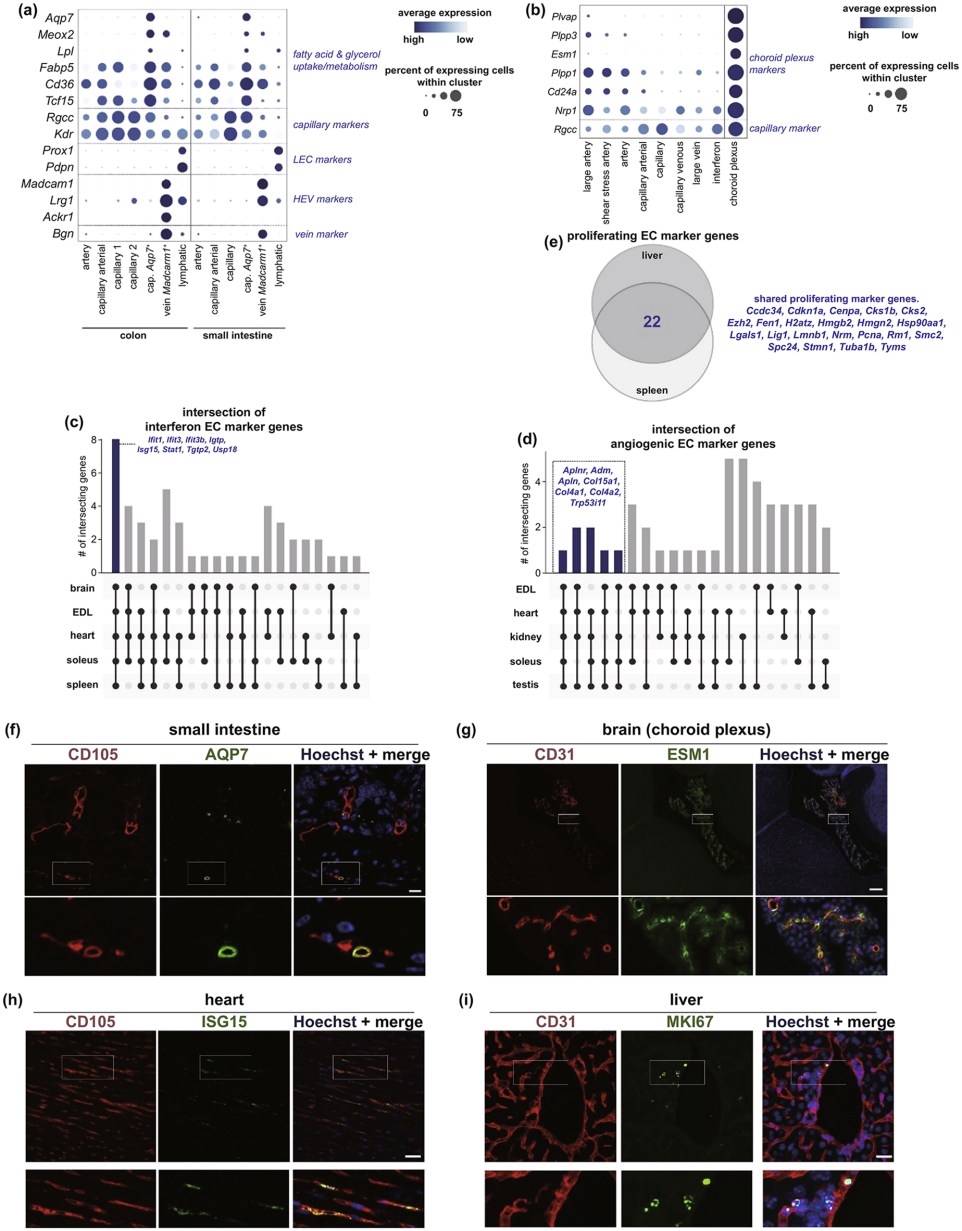
Specialized and unexpected endothelial cells phenotypes. **a**, **b** Dot-plot heatmap of markers enriched in intestinal *Aqp7*^+^ capillary and *Madcam1*^+^ vein ECs. **c**, **d** UpSet plot of intersections between the top 50 markers expressed by interferon-activated. **e** Venn diagram showing genes upregulated in proliferating ECs. **f**–**i** Representative micrographs of mouse tissue sections. Reprinted from [95] with permission

**Fig. 8:**
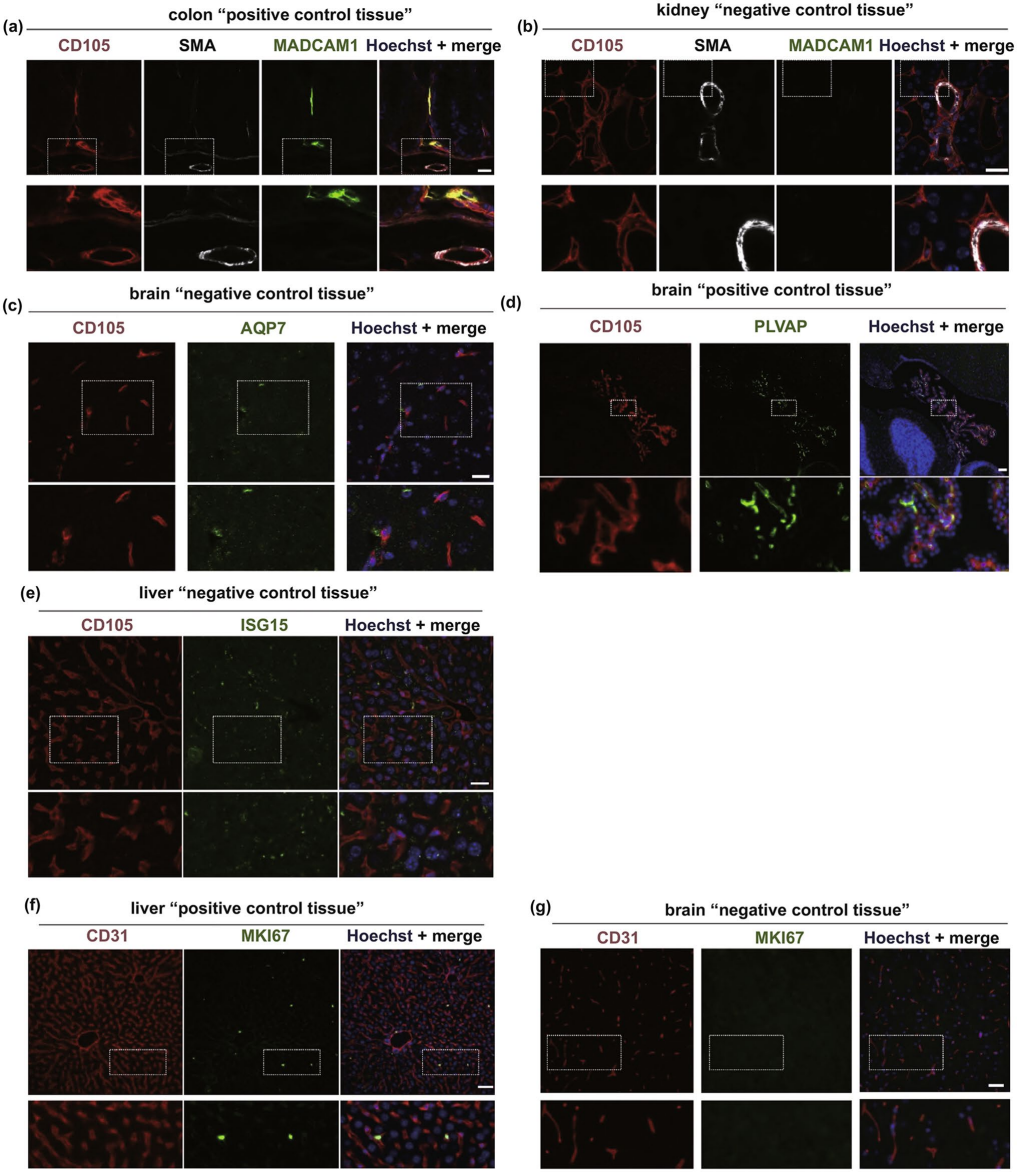
Protein validation of specialized endothelial cells phenotypes. **a**, **b** Representative micrographs of mouse colon. **c**, **d** Representative micrographs of mouse brain sections (negative and positive control group). **e–g** Representative micrographs of mouse liver (negative and positive control groups) [95]

In many bioengineering strategies, researchers have resorted exploiting primary endothelial to fabricate meso- and microscale vasculature [89–91]. Co-bioprinting of human umbilical vein endothelial cells and primary mouse hepatocytes presented promising cell–cell interaction and significantly enhanced the metabolic activity of CYP1A2 and the functionality of hepatocytes due to proper induced vascularization [92]. Bersini et al. reported that their 3D bioprinted human muscle-specific endothelium model is differentiated from primary endothelial cells and is supported by a vascular network [93]. Recently, critical role of endothelial cells in hair growth has been disclosed, in which an aggregate made of human vascular endothelial cells, a human dermal papilla suspension and mouse embryonic epithelial cells was formed, which then localized in hair follicle germs; system improved follicular gene expression leading to hair regeneration [94]. Carmeliet et al. published a single-cell study revealing the transcriptome atlas of endothelial cells that provides a better characterization of endothelial cells heterogenicity which may solve the main challenge to simulate the vascular models in micro- and mesoscales, especially in regenerative medicine. This atlas contains the whole transcriptome of endothelial cells in both specialized and unexpected (Fig. 7) endothelial phenotypes. All of these results have been validated by protein–protein investigations as well as the specific protein analysis (Fig. 8), which could help the scientists to evaluate the behavior and interaction of the nanomaterials and their interconnected relationships inside the cellular microenvironments [95].

There is a critical role for pericytes in autocrine and paracrine signaling to affect the microvasculature. Besides, the capillary formation and regeneration is regulated by pericyte abundance, specification and plasticity [96]. Some challenges have been encountered in recapitulating these cells in vitro; neural-glial antigen 2, smooth muscle actin and platelet-derived growth factor receptor-beta are surface markers expressing in different varieties that can create a potential to be differentiated into smooth muscle cell and other stromal cell types [97]. Perivascular cells have the potential to trigger the factors impacting to supply some of the supportive scaffolding for angiogenic sprouting formation in microvascular models, although they lack the tissue-specific hallmarks of pericytes. Consequently, perivascular cell replacement as a critical bioengineering approach involves differentiating stem cells (i.e., bone marrow or primary fibroblasts-derived MSCs) in engineered models [98–101]. For example, researchers reported human-derived MSCs and endothelial cells interaction under controlled perfusion circumstances provided by microfluidic device which has resulted in promotion of MSCs proliferation, and proper response of the vascular cells [102]. Composite microbeads fabricated by fibrin and collagen embedded with human fibroblasts and endothelial cells showed pericyte-like function, and deposition of laminin that is indicated in the microvessel network maturation over 14 days in vitro culture and formation of prevascularized microtissue [103]. In another study, collagen microspheres have been loaded with human bone marrow-derived MSCs to stimulate the pluripotent stem cell-derived endothelial cells (iPSC-EC). Herein, due to MSCs interaction with iPSC-EC, they adopted a phenotype of an endothelial cells, expressing CD31 that is the endothelial marker, forming tubular microvessel-like patterns [104].

**Fig. 9:**
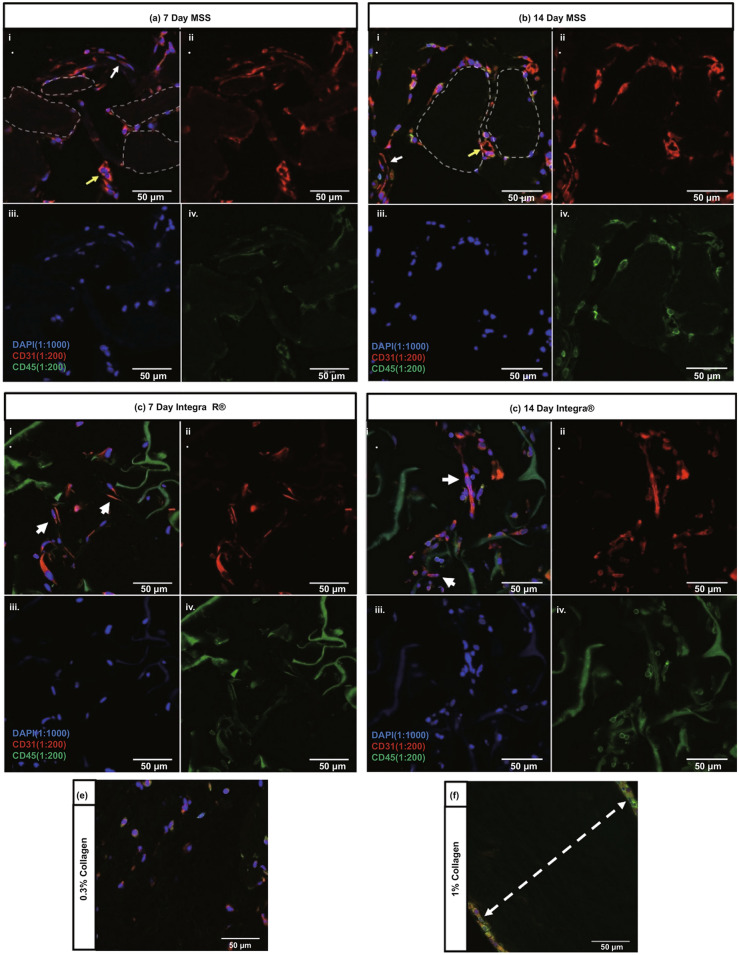
In vivo phases on the neovascularization’s experiments; **a** CD31 and CD45 stainings of microsphere scaffolds; **b** endothelial cell stainings of microsphere scaffolds; **c**, **d** endothelial cell stainings of Integra® scaffolds; **e**, **f** CD31 and CD45 stainings of type I collagen scaffolds [111]

**Fig. 10:**
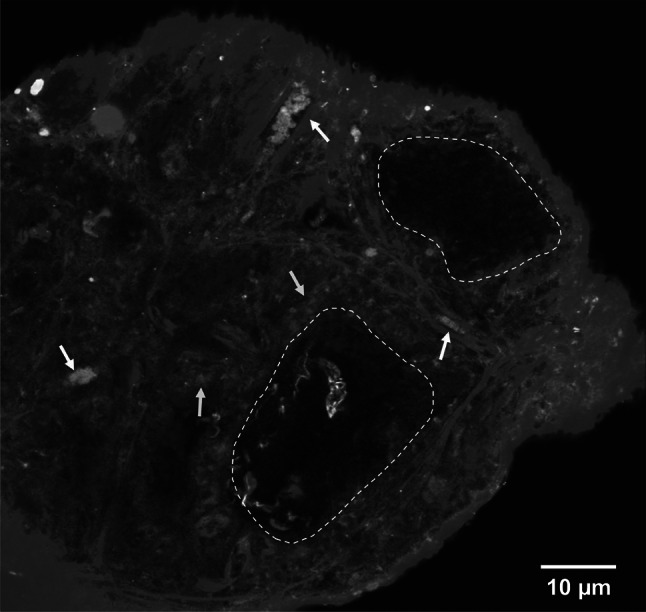
Multiphoton microscopy of the stained microsphere scaffolds after 14 days [111]

Employing novel cell sources including iPSC tactics for microvascular tissue engineering or in vivo regeneration have been greatly discerned. The derivative methods of iPSC-human endothelial cells (ECs) (CD144 + , CD31 + antigen expression) generate arterioles or venous-like endothelial populations [105–108]. Compared to HUVEC, some studies have emphasized the lack of endothelial maturation to induce functional deficits after ectopic implantation in the body [89]. A recent study by the Levenberg group indicates the ability of endothelial cells (ECs) which are derived from embryonic stem cell (ESC) to anastomose into native murine tissue, as demonstrated by the first case of human CD31 + blood vessel cells in the lumen [109]. Early work revealed CXCL12/CXCR4 chemotactic signaling in iPSC-ECs can promote revascularization of the ischemic retina which contributed significantly to vascularization better than human primary endothelial cells [110]. Collagen-based microspheres loaded with human umbilical vein endothelial cells (HUVEC) have been developed and showed remarkable increased of CD31 + suggesting neovascularization after 14 days in vivo implantation (Fig. 9); results have been supported for the first time by multiphoton microscopy with high-resolution spectra (Fig. 10) [111].

Diverse approaches are available to direct differentiation protocols for pericytes and generation of iPSC- and ESC-derived perivascular cells as has been reported by Gerecht and colleagues [112]. ECs, pericytes and vascular smooth muscle cells (VSMCs) can now be generated with high efficiency from human-induced pluripotent stem cells (hiPSC) and used in organ-on-chip development as a fundamental in vitro vascularized model to mimic tissue [113]. In addition to in vitro models, many groups have attempted to obtain functional in vivo implantation in regenerative injury models [114, 115]. The use of iPSCs in engineering meso- and microscale vasculature is an autologous and still unlimited cell source as well as high yield technique affording a most effective tool to generate tissue vascularization and in vitro models.

## Gaps and Role of Bioprinting to Address Issues

Advanced techniques have emerged in microvascular tissue fabrication, including extrusion-based [116, 117] and droplet-based bioprinting [118], Kenzan [119], and biogripper approaches [120, 121] leading to 3D bioprinting of cell-laden aggregates and spheroids.

Extrusion-based bioprinting is a powerful 3D technique that distributes a bioink solution through a nozzle and such bioprinting of cellular aggregates can be used for cell aggregation and tissue strand maturation to generate scalable tissues [122]. As an example, Mao et al. encapsulated human-induced hepatocytes (hiHep cells) within a bioink made up of methacrylated gelatin (GelMA) and liver decellularized extracellular matrix (dECM) to form liver microtissue; significant secretion level of albumin and blood urea nitrogen has been reported indicating excellent liver function [123]. However, most of the printable bioinks comprise similar-sized spheroids suspended in a hydrogel may self-aggregate inside the nozzle during the printing process and therefore clog the needle [124]. The development of organs-on-chips and micro-physiological systems utilizing extrusion-based bioprinting technology requires highly precise technical details. A comprehensive review on microtissue biofabrication using extrusion-based bioprinting is available [125].

**Fig. 11:**
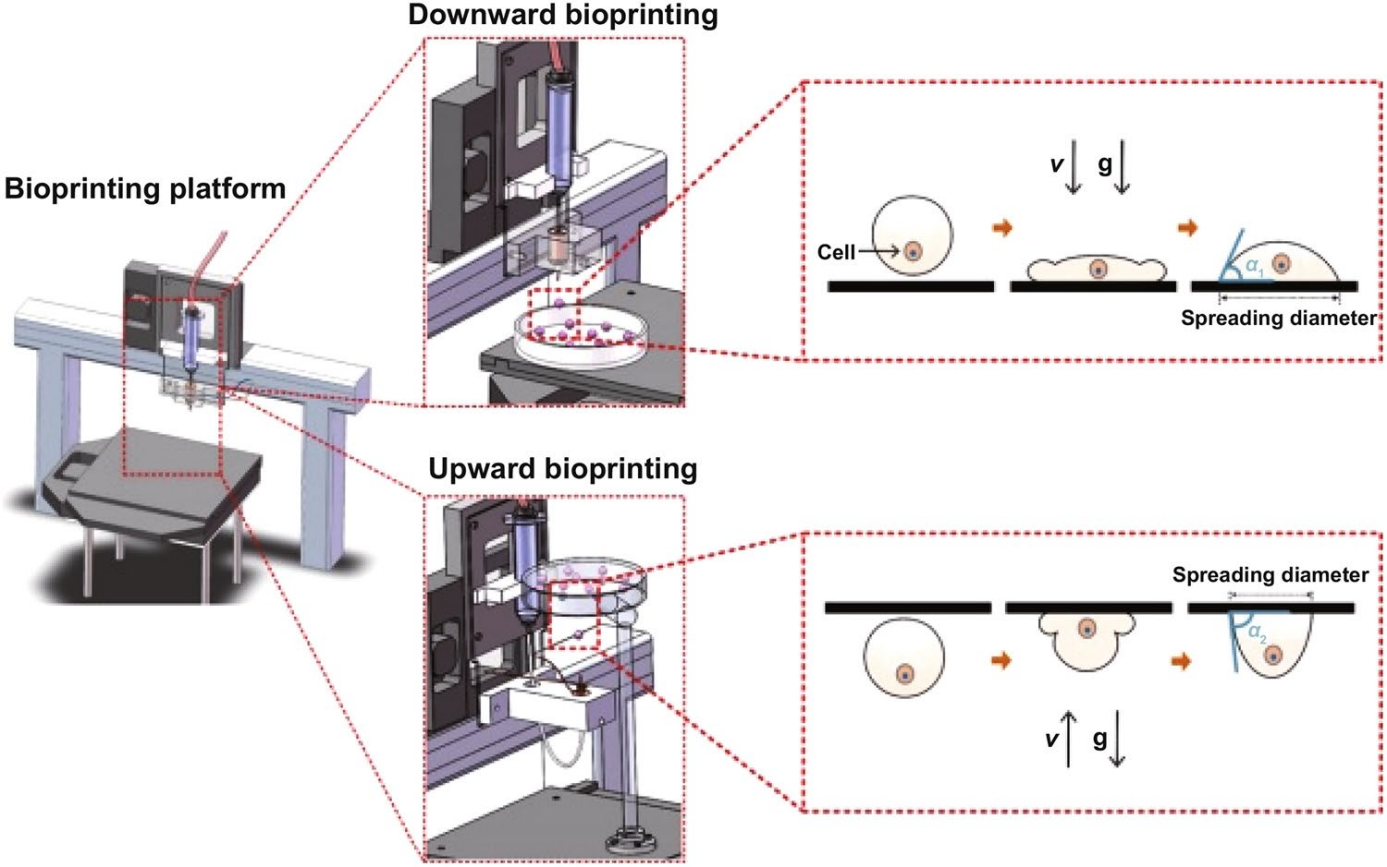
A schematic illustration of the upward and downward bioprinting devices, as an advanced and high-resolution droplet-based bioprinting systems. Reprinted with permission from [130]. Copyright 2021 American Chemical Society

Droplet-based bioprinting (DBB) use a bioink solution to generate biomaterials droplets and cells in high precision. Droplet-based techniques can be further classified into inkjet bioprinting, microvalve bioprinting and acoustic-droplet-ejection bioprinting [126–129]. Droplet-based bioprinting is appropriate for printing microvasculature for high‐resolution patterning in the diameter range of 50 − 300 μm (Fig. 11) [126, 130, 131]; presently, it enables precise positioning of spheroids in 2D, in which spheroids are loaded in a droplet during bioprinting [132]. The formation of the capillary network in the DBB method is based on the self-organization of ECs in printed biological materials. Therefore, the bio-paper substrate is an ideal choice for promoting the formation of microvessels after bioprinting. Biological material plays a critical role in microvasculature bioprinting. It is therefore necessary to suppose the proangiogenic properties of both the bioprinting substrate and the bioink; ability of the substrate and bioink unite to promote microvasculature formation. The functional formation of vascular networks depends on rapid self-assembly and high printability structures. Benning et al. introduced an evaluation of commonly used hydrogel bioinks as they demonstrate that fibrin and collagen hydrogels have the potential to provide the necessary support for the human umbilical vein endothelial cells (HUVEC) proliferation in 2D and the capacity to sprout from HUVEC spheroids after 3D printing [133]. The precise spatiotemporal control is presently limited to nano- or picoliters in volume that allows biomaterials, cells, and other biologics deposited in a very precisely controlled manner. In this regard, Boland’s group established modified commercial printing technology for the direct droplet-based bioprinting of microvasculature. The modified inject bioprinter deposited smooth muscle cells and bovine aortic endothelial cells onto collagen and matrigel, respectively [134]. The cell viability was achieved after 3 days of in vitro culture.

Spheroids and microtissues with various biological compositions, e.g., different cells, could not be deposited in arbitrary positions while maintaining spheroids in predetermined positions is challenging. To address this problem, 3D deposition of spheroids and microtissues using microneedles have been introduced [121]. A commercial device capable to bioprint spheroids in a precise way has not been available until the invention of Kenzan method which is a microneedles-based technology launched in Japan [135, 136]. This array of needles bioprint cell-laden spheroids into predesigned contiguous temporary support with micron-level precision [137]. Then, the spheroid-based structure is cultured until the spheroids fuse into cellular aggregates and synthesize their own ECM [138]; spheroids generated using this method have uniform shapes and sizes. During insertion, smaller spheroids have the potential to disintegrate on the needle [137]. To biofabricate microvessels, Kenzan technology has been used, in which human dermal fibroblasts (hDFs) are loaded in spheroids and create tubular structure. Several cells type like vascular endothelial cells and vascular smooth muscle cells migrate into the structure after transplantation of the structure in pigs [139]. Zhang et al. reported study of diaphragm-like patch using Kenzan method. Tubular structure is developed by positioning of hDFs and hUVECs-laden spheroids. Results of in vivo transplantation in rats showed blood vessels and nerve regeneration [140].

Another technology that has been introduced is biogripper method that is capable of fabricating large perfusable tissue structures through positioning, aligning and manipulating scaffold-free bio-microaggregates with wide range of sizes (600 µm to 3.4 mm) and shapes. The micro-sized aggregates are fragile; therefore, it is highly demanded to keep them in an aqueous environment [141, 142]. Recently, Blanche C Ip et al. introduced a modified and flexible biogripper system with improved optical transparency for the precise alignment of micro-sized aggregates. The force-driven fluid is engineered to deposit aggregates without inducing any damage to the cells [142].

## Micro-sized Aggregates and Microspheres/Microbeads

Creating a variety of different cell formations including large-scale spheroids [143], honeycombs [141], and strands [144] has been achieved by multiple cell types and co-culture of aggregated cells. The ability of extracellular matrix secretion constitutes a significant point for the cells which induce effective communication in a native-like microenvironment [72, 123, 145]. Native-like tissue microenvironment usually is not facilitated due to the limited cell–cell and cell–ECM interactions. It can be attained by the growth of cells in an isolated fashion such as cell-laden hydrogels or cells in monolayers [146]. To address the issue, novel bioprinting technologies have been introduced. Many bioinks formulations including cell aggregates have been reported as a promising tool for bioprinting vascularized tissues. Moreover, its ability for precise placement of high-density cells in the desired location creates a biomimetic construct at a clinically applicable scale. As an example, in a functionalized and high survival of 3D culture model of pancreatic islets, microvascularization is highly demanded. Scheiner et al. employed 50-μm poly(ε-caprolactone-PEG-ε-caprolactone)-b-poly(l-lactide) microspheres laden with VEGF in 3D printed poly(dimethylsiloxane)-based construct and then loaded with the islet cells, which resulted in significant vascularization within 4 weeks [125].

One of the main challenges in vascular tissue engineering involve the lack of cell–cell interactions and microvasculature network sprouting in the cell culture systems which can be addressed by generation of cell-laden spheroids or micro-sized aggregates. Due to the ability of spheroids to fuse, they can be used to form robust endogenous capillary-like networks which can offer new opportunities for creating highly organized vascularized microtissues [147]. In this regard, it has been shown that sprouting of capillary angiogenesis is capable of changing mechanobiological properties of a fibrin cell-laden matrix altering cell fate [148, 149]. In another study, agarose micromolds are used as high yield spheroids with controlled dimensions to develop a platform for high-throughput fabrication of prevascular networks [150]. HUVECs can be successfully differentiated into spheroids and have been recently used in combination with adipose-derived stem cells (ADSCs) to improve the capillary formation within the spheroids in vivo and tissue engineering applications [151, 152]. The laser-induced forward transfer (LIFT) process is a prospective digital printing technique that utilizes hydrogels with a viscosity ranging from 1 to 300 mPa s to promote droplet formation which may lead to a higher cell density (up to 60 million cells mL^−1^) in the LIFT printing technique [153]. Wu and Ringeisen fabricated capillary-scale branch/stem structures of human umbilical vein endothelial cells (HUVEC) similar to the vein structure on a leaf using biological laser printing (BioLP) [154]. Recently, a LAB bioprinter (laser-assisted bioprinting) was advanced for patterning of endothelial cells in situ [155]. The approach of this method was to develop organized vascular networks due to the cell self-assembly, which subsequently lead to increased bone tissue regeneration by enhancement of vascularization in engineered constructs. The comparison between randomly seeded endothelial cells and cells printed onto a collagen substrate containing human VEGF and MSCs, provided evidence for the applicability of LAB in clinical applications. The vascularized constructs method has been used to print smooth muscle cells and HUVECs sequentially adding into a branched structure 2D configuration [156].

In another study, bioinks based on thrombin and calcium have been deposited on a surface of fibrinogen to simulate the intrinsic solidifying process during wound healing [157]. This study established a dry inkjet printing method with in situ biomimetic gel formation where the printed fibrinogen exhibits explosive pressure that can model cell microvessels in two dimensions (2D) and form stable channels within 21 days [157].

Microtissues in the form of tissue spheroids and other small cell aggregates in 3D are ideal candidates to simulate tissue microenvironments in vivo, which can be reconstituted to generate reproducible complex tissues such as bone [158] and pancreas [159] and cancer tissue models for therapeutic purposes [160]. The assembly of this kind of spheroids can occur in a controlled manner by 3D bioprinting and microfluidic devices [160–162]. Therefore, these tissue spheroids must have a standard size and shape suitable for the continuous dispensing in the microtissues bioprinting process. Chen et al. biofabricated in vitro human 3D vascular cancer model where perfusion and microcirculation of tumor cells are tracked and monitored over time through confocal microscopy [161]. Lim et al. have recently reviewed microvascularized aggregates as a various 3D cancer models for drug screening [163].

The scalable biofabrication of a large volume of standard size is another essential issue that should be considered [164]. Large spheroids with a diameter (> 300 μm), or smaller co-culture spheroids are non-compatible for bioprinting. Hanging drop culture is an example of high maintenance culture methods that leads to small co-culture spheroid formation [165]. The suitability of vascularized spheroids formation has been investigated by several culture methods [166, 167]. Despite the development of multiple high-throughput spheroid culture systems, so far they have been used to produce monoculture spheroids [127] or non-vascularized co-cultures [148].

A method to produce prevascularized micro-sized aggregates and microspheres with tunable and controlled size which enables the formation a macrotissue in vivo in high yield still has not been reported. Furthermore, these prevascularized microspheres will be essential elements for 3D bioprinting of microtissue which is mimicking the particular human tissue histoarchitecture. To date, most of the engineered vascularized tissues are in range of 1 mm in size. However, microvascularization is required in large with high cell density (~ cm) to provide proper perfusion [119]. Moreover, the non-adherent microwell culture system can generate prevascularized microtissues with bioprinting‐compatible geometry in a high-throughput manner [168]. Co-culture of human umbilical vein endothelial cells (HUVEC) with other supporting cell types such as adipose tissue-derived mesenchymal stem cells (ADSC) and human foreskin fibroblasts (HFF) in different ratios (HUVEC/ADSC, HUVEC/HFF, HUVEC/ADSC/HFF) are used to study the impact of applied supporting cell types and cell ratio on spheroid and microvessel formation. The ability of the spheroids in random self-assembly into a larger macrotissue has been studied. Spheroids loaded with endothelial cells exhibited enhanced generation of a denser tissue microenvironment, inherent ECM secretion, and prevascularized network [169]. Overall, larger and complex vascularized tissue structures are made using prevascularized cell aggregates as cardinal building units which lead to precise control of representations of native tissues [166, 170]. Furthermore, these tissue complexes can be more appropriate than conventional animal models used for drug assessment, disease monitoring, and high-throughput screening [171].

Tissue spheroids are 3D living materials with certain measurable, and controllable composition, material, and biological properties. Enabling precise bioprinting of various shapes of spheroids onto functional gel substrates (i.e., alginate) has been demonstrated. Aiming to obtain fibrin constructs, the microvalve bioprinting has been used to print fibrinogen and thrombin in the form of microspheres, in which spheroids are embedded into the fibrin as a functional hydrogel. MSC/HUVEC spheroids with average diameter of 300 µm are then bioprinted into desired positions within the fibrin hydrogel before the constructs are fully cross-linked [154].

In another study, the matrix of spheroids is made of HUVEC, 3t3, and 4t1 cells in the diameter range of 80 to 200 µm. Eight MSc spheroids in different sizes were bioprinted, on top of each other without any gel support, to fabricate a hollow bridge shape for confirming the precise positioning control of aspiration-assisted bioprinting (AAB) [172]. Moreover, spheroids could be bioprinted into sacrificial hydrogels or functional hydrogels sequentially as scaffold-free bioprinting or scaffold-based bioprinting. Using this strategy, the bioprinting of non-uniform sized spheroids is enabled.

Self*-*assembled vascular microtissue spheroids allow large and complex vascular tissue engineering. For example, et al. showed microvascular tissue formation from entrapped cells within an RGD-modified alginate-based microgel. Microgel modification tuned the mechanosensing and ECM regulation resulting in microtissue self-assembly [161]. To develop the hollow vascular spheroids, Gentile et al. studied VEGF-mediated fusion in the generation of embryonic mouse allantois-derived spheroids; analysis of VEGF-treated spheroids showed that treated spheroids are uniluminal [173]. This method produced the transformation of the inner network of small diameter endothelial tubes into a contiguous layer of cells that circumscribed a central lumen-like cavity. These uniluminal spheroids can promote formation of blood vessels and microvasculature while still retaining their hollow core [174].

The coating of spheroids with ECs is another strategy to fabricate vascularized macrotissues. Microtissues are coated with primary endothelial cells that can assemble to form macrotissues with endogenous vasculature [175]. The presence of induced pluripotent stem cell-derived endothelial cells on the surface of microbeads promoted the microvascular network sprouting and angiogenesis in a hepatic cell-dense 3D tissue [176]. It has been reported that in vivo implantation of vascular spheroids based on human endothelial cells leads to the significant production of blood microvessels and lymph [177]. Angiogenicity of dental pulp stem cells and endothelial cells self-assembly into capillaries led to increased blood perfusion in the implanted collagen-based scaffold [178]. Scientists showed that the anastomosis prevascularized endothelial-fibroblast aggregates generated microcapillaries resulting in high post-implanted cell viability [179], though further studies are required in large animal models to confirm potential of the above-discussed microtissues for the clinical applications.

Despite the advances, the architectural complexity of vascularized micro- and macrotissues is still challenging. The approach of scaffold-free vascular tissue engineering provides the possibility of designing vessels with various shapes and diameters. This method is fast, accurate, reliable and easily scalable [180]. Regarding this issue, bioprinting technology has been used to develop scaffold-free cell-laden construct. Jakab et al. studied 3D biofabrication of the ring and tube-like structures by 3D deposition of Chinese Hamster Ovary (CHO) cells-laden microspheres, leading to tunable cell–cell interactions and adhesion by varying hydrogels properties [181]. In addition, the embryonic cardiac cell aggregates and ECs-loaded structures have been bioprinted for cardiac tissue engineering, in which the ECs migrate to the space between aggregates allowing cell–cell interaction [182]. Due to cell–cell interaction provided proper ECM formation, injectable pre-cultured HUVEC and normal human lung fibroblasts (NHLF) cell-loaded fibrin microbeads present an excellent in vivo microvascularization in ischemic site after 7 days [183]. Tan et al. proposed and developed ring-shaped molds with alginate microdroplets. Spheroids comprising human aortic smooth muscle cells (hSMCs) and human umbilical vein endothelial cells (HUVECs) are deposited onto an alginate hydrogel substrate [164]. The spheroids fusion increased the levels of formation of toroid-shaped tissue and lead to endogenous collagen secretion during in vitro culture. Alternatively, using an alginate-based bioink to bioprint cell aggregations within calcium chloride solution for crosslinking, enables the fabrication of zigzag cellular tubes structure [184].

Precise positioning of spheroids is a significant challenge. Spheroid density has a crucial role in the spheroid fusion [185]; therefore, the inhomogeneous fusion may ensue due to the inconsistent placement of spheroids [186]. In this regard, the spheroids are perfused into solid cylinders before printing. Cylindrical tissue unit fusion is faster and more continuous especially on large scale in comparison with spherical units [187]. These structures can be used for scale-up vascular tissue biofabrication [180].

Several types of vascular cells including smooth muscle cells and fibroblasts that have accumulated into various components lead to engineer multi-cellular microtissue [188]. The multicellular spheroids or tubes with average diameter of 300 to 500 µm have been bioprinted using agarose rods as non-adherent mold. The combination of different cells made it possible to obtain single-layer or double-layer blood vessels [189, 190]. Maturation factors can activate endothelial cells, fibroblast and MScs which are micro-structured by layer-by-layer bioprinting on the hydrogel. This process leads to vascularization, which is confirmed by the gene expression in endothelial cells [191]; further details are available in a recent review by Sarker et al. [192].

## Conclusion and Future Outlook

Studies on vascular tissue engineering are growing rapidly and can be further improved using advanced technologies such as 3D bioprinting and microfluidic devices. Traditionally, the growth factors and angiogenic progenitors are incorporated into the scaffold to increase cell infiltration and vascularization. However, the biological response is limited, particularly in large and cell dense tissues. It is therefore preferred that the microvascularized systems used in the engineered construct allow proper cell–cell and cell–ECM interactions. Prevascularized micro-sized aggregates or microspheres enhance the vascularization potential of the structures and save the time required for angiogenicity to advance within the scaffold. However, precision deposition of microvascularized spheres within the scaffold and clinical application of such prevascularized scaffolds are still challenging. Recently, comprehensive clinical trials that are underway or planned in the near future seem to show that we are entering a new era of vascular disease treatment.
